# More than meets the eye: understanding *Trypanosoma brucei* morphology in the tsetse

**DOI:** 10.3389/fcimb.2013.00071

**Published:** 2013-11-13

**Authors:** Cher-Pheng Ooi, Philippe Bastin

**Affiliations:** Trypanosome Cell Biology Unit, CNRS URA2581, Institut PasteurParis, France

**Keywords:** trypanosome, tsetse, life cycle, cytoskeleton, morphological changes, stage-specific regulation

## Abstract

*T. brucei*, the causative parasite for African trypanosomiasis, faces an interesting dilemma in its life cycle. It has to successfully complete its infection cycle in the tsetse vector to be able to infect other vertebrate hosts. *T. brucei* has to undergo multiple morphological changes as it invades the alimentary canal of the tsetse to finally achieve infectivity in the salivary glands. In this review, we attempt to elucidate how these morphological changes are possible for a parasite that has evolved a highly robust cell structure to survive the chemically and physically diverse environments it finds itself in. To achieve this, we juxtaposed the experimental evidence that has been collected from *T. brucei* forms that are cultured *in vitro* with the observations that have been carried out on tsetse-infective forms *in vivo*. Although the accumulated knowledge on *T. brucei* biology is by no means trivial, several outstanding questions remain for how the parasite mechanistically changes its morphology as it traverses the tsetse and how those changes are triggered. However, we conclude that with recent breakthroughs allowing for the replication of the tsetse-infection process of *T. brucei in vitro*, these outstanding questions can finally be addressed.

## Introduction

The life cycle of *Trypanosoma brucei* within the tsetse is one that is complex and spectacular. The changes observable in *T. brucei* as the parasite progressively invades the tsetse vector have been documented for decades, yet recent discoveries have added even more threads to an already fascinating tapestry.

In this review we will attempt to bring together two seemingly disparate fields: the elucidation of the *T. brucei* life cycle within the tsetse, and the understanding of the parasite from a viewpoint of cell biology, to better understand how the parasite mechanistically alters its morphology while infecting the tsetse. To discuss such a complex topic, it is inevitable that we first have to establish a backdrop against which these issues are discussed by narrating the characteristics of *T. brucei* and the intimate relationship it shares with its insect vector.

### African trypanosomes

African trypanosomes are protozoan parasites that are the causative agents of human African trypanosomiasis (HAT) and animal African trypanosomiasis (AAT). Both the human and animal forms of trypanosomiasis are fatal without treatment. Due to an increase in disease monitoring and treatment, HAT is currently in its decline since reaching a peak in the 1990s (Simarro et al., 2011). However, the incidence of cases can still be high in rural areas which lack continuous surveillance programs (Chappuis et al., 2010) and history has likewise amply demonstrated that decline is not necessarily a prelude to eradication (Simarro et al., 2008). Therefore, it is premature to write off HAT as a public health concern in sub-Saharan Africa. Furthermore, AAT causes an ongoing agricultural dilemma in Africa as it restricts the rearing of meat- and dairy-producing livestock while depriving farmers in endemic areas the use of draught animals. Both the human and animal disease combined still constitutes a major hindrance to the development of the African continent.

African trypanosomes are characterized by a single flagellum and a compact disc of mitochondrial DNA termed the kinetopast. They have co-evolved quite extensively with their tsetse vectors. As such, African trypanosomes have developed life cycle stages of varying complexity to allow for transmission by the tsetse. Evidence of this co-evolution include the complexity of the kinetoplast genome, which has been found to be eroded in trypanosome species which have relatively recently escaped the dependence on tsetse for transmission (Lai et al., 2008). This is further lent credence when laboratory manipulation to produce parasites that have either partially or completely lost their kinetoplast usually results in parasites that remain viable, but are locked in their bloodstream form (BSF) stage (Lai et al., 2008) and thus unable to infect tsetse. Furthermore, the various canonical tsetse-specific morphologies of the parasite are localized to defined organs within the tsetse, suggesting that these forms were evolved specifically to overcome the challenges associated with traversing their insect vector.

Besides interest in them as causative agents of disease, African trypanosomes, more specifically *T. brucei*, have received much attention as model organisms with which a multitude of questions related to fundamental cell biology can be answered. The availability of a fully sequenced genome and various molecular tools make it feasible to generate loss of function and gain of function cell lines in *T. brucei*. Furthermore, a multitude of molecular probes have been developed for the observation of molecular changes in those cell lines. This has led to many new insights in molecular biology having been discovered in cultured *T. brucei*. This includes the discovery of RNA editing (Benne et al., 1986), base J (Gommers-Ampt et al., 1993) and glycosylphosphatidylinositol (GPI) anchors (Ferguson et al., 1988). In a more medically relevant context, the single flagellum in *T. brucei* makes it an ideal model organism for understanding the genes related to various ciliopathies in humans, as the genes encoding structural and constructional elements of the parasite flagellum are likewise conserved in humans (Branche et al., 2006; Baron et al., 2007).

### The *T. brucei* cell structure

**Figure 1:**
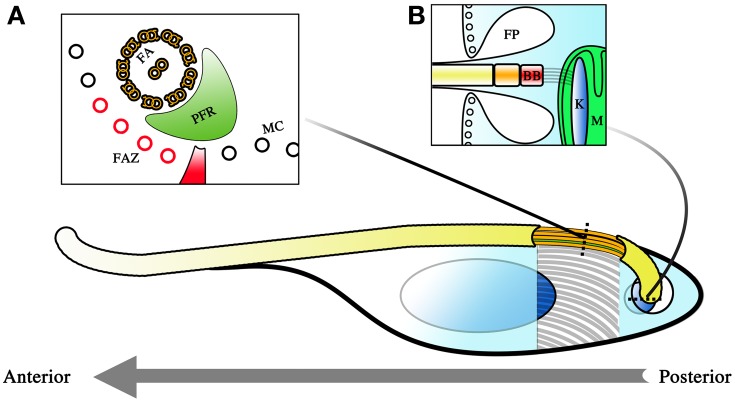
**Diagram representing the cellular architecture of *T. brucei***. The parasite is a unicellular kinetoplastid with a single flagellum. The polarity of the cell is determined by the polarity of the microtubules comprising its cytoskeleton, with the positive pole as its posterior and its negative pole as its anterior. This is also the direction in which the flagellum extends and the primary direction of motility. Beneath the membrane, a microtubule corset (MC) gives the parasite its cellular shape **(A)**. The flagellum winds its way toward the anterior of the cell, and is given form by the flagellar axoneme (FA) and the paraflagellar rod (PFR). The flagellum attachment zone (FAZ, colored red), which comprises of a specialized quartet of microtubules and a filament, lies just beneath the flagellum on the MC. The polarity of the FA and FAZ microtubules is with the positive end away from the reader while the MC has its positive end toward the reader. The flagellum exits the cell body at the flagellar pocket where an invagination of the membrane is formed **(B)**. This flagellar pocket (FP) is free from the spatial constraints of the microtubule corset, and is the only site for endo- and exocytosis within the cell. At the base of the flagellum is the basal body (BB), and beneath that, the kinetoplast (K), which is a compact disc of mitochondrial (M) DNA. The BB and the K form a single connected entity, with the BB tethered to the K by filament structures. Corset elongation only occurs at the posterior end of the cell where tubulin incorporation into the cytoskeleton allows for extension of the cytoskeleton. This occurs either as part of the cell cycle or as part of morphological changes which occur during infection of the tsetse.

*T. brucei* has a robust yet flexible cellular structure (Figure 1). A microtubule corset forms a tight “cage” that defines the shape of the cell (Figure 1A). This corset structure is highly resistant to damage and is possibly a protective adaptation to help the parasite survive in environments that are highly variable in their chemical or physical characteristics.

The robustness of this corset and its role in maintaining the morphology of the parasite is most obvious in detergent extraction experiments when cellular membranes are stripped. Even with only the cytoskeleton remaining, the shape of the cell is still maintained (Sherwin and Gull, 1989). So tightly spaced are the microtubules of the cytoskeleton that it limits access to the cell body. Membrane invagination to facilitate endo- and exocytosis is therefore impossible along most of the cellular surface. As such, molecular exchange into and out of the cell body is only possible at the flagellar pocket (FP) where the flagellum exits the cell body (Figure 1B). Since the flagellar membrane maintains continuity with the rest of the plasma membrane, this exit point creates a small area of membrane surface that is unhindered by the physical constraints of the microtubule scaffold and is thus free for transfer of materials to and from the cell. Endocytosis is of major importance in BSF stage trypanosomes where receptor-mediated endocytosis is a matter of survival (Allen et al., 2003; Engstler et al., 2004). This is because essential processes such as the turnover of the variant surface glycoprotein (VSG) coat is dependent upon endocytosis. Endocytosis is thought to be of lesser importance during the procyclic trypomastigote stage (Garcia-Salcedo et al., 2004), though these observations have only thus far been carried out in cultured procyclic trypomastigotes and may not reflect the situation in the tsetse.

Within this microtubule framework sit the various single copy cellular organelles (when considering a non-dividing cell) that make *T. brucei* such an attractive model organism for investigations into cell biology. The kinetoplast is located close to the base of the flagellum near the FP, even though the mitochondrion extends the entirety of the cell body and is even highly branched in the case of tsetse-infective forms. Cells with a kinetoplast that is posterior to the nucleus along the anterior-posterior axis of the cell are termed trypomastigotes while cells with the kinetoplast anterior to the nucleus are termed epimastigotes. The basal body (BB) is located at the base of the flagellum and is tethered to the kinetopast via filament structures that traverse the cytoplasm and both mitochondrial membranes (Ogbadoyi et al., 2003).

The parasite has a single flagellum that drives locomotion toward the direction in which the flagellum elongates. *T. brucei* motility is therefore best described as a dragging movement of the flagellum, quite different from the propulsion of many flagellated systems. The BB has a 9 + 0 triplet microtubule configuration that then extends as doublets in the transition zone. A central pair is added on top of the structure to yield the 9 + 2 microtubule doublet configuration that is the hallmark of motile flagella. The majority of the organelle is attached to the cytoskeleton of the cell body via a region known as the flagellum attachment zone (FAZ). The FAZ is comprised of four specialized microtubules and a filament (Sherwin and Gull, 1989). The flagellum thus extends toward the anterior of the cell in a left-handed helical manner adhered to and coiled around the cell body. The distal end of the flagellum is detached from the cell body, forming an overhang over the anterior portion of the cell. Another peculiar feature present in the trypanosome flagellum is the paraflagellar rod (PFR), a crystalline structure that spans the majority of the parasite flagellum. Although important in maintaining the beating of the flagellum (Bastin et al., 1998), its precise function remains unknown. The flagellum of the parasite is constructed by a phenomenon called intraflagellar transport (IFT), best described as a circuit of cargo trains trafficking from the base of the flagellum to its tip (Kohl et al., 2003; Absalon et al., 2008).

The consistency of the microtubule corset that makes up the cytoskeleton is not uniform across the entirety of the cell. The posterior end of the cell is a region where elongation of the microtubule corset takes place. This region is characterized by the localization of tyrosinated α-tubulin. Newly synthesized α-tubulin is tyrosinated and this tyrosination is gradually lost over time (Sherwin et al., 1987). Antibodies specific for tyrosinated α-tubulin can thus be used to observe the localization of newly assembled cytoskeletal microtubules. This method has been effectively exploited to study the assembly and elongation of the cytoskeleton *in vitro* (Matthews and Gull, 1994; Matthews et al., 1995), the morphological changes for trypanosomes in the tsetse (Sharma et al., 2008; Rotureau et al., 2011), as well as the cellular changes invoked during ectopic expression of regulatory factors relating to *T. brucei* cellular elongation (Hendriks et al., 2001; Li and Wang, 2003; Hammarton et al., 2004). Besides the posterior end of the cell, some tyrosinated α-tubulin has been observed to co-localize with the BB of the mature flagellum (Stephan et al., 2007).

The cellular membrane of *T. brucei* is anchored to this cytoskeleton scaffold by microtubule-associated proteins (MAPs). These proteins can have other functions, such as maintaining the spacing between microtubules in the cytoskeleton (Robinson et al., 1991). Furthermore, MAPs such as CAP5.5, CAP15, CAP17, and TbAIR9 are linked to maintaining the stability of the cytoskeleton (Hertz-Fowler et al., 2001; Vedrenne et al., 2002; Olego-Fernandez et al., 2009; May et al., 2012). Distribution of these proteins across the cell is not homogenous, and their expression and sub-cellular localization is cell cycle stage dependent. Perhaps unsurprisingly CAP15 and CAP17, the cytoskeleton-stabilizing MAPs have been observed to be less abundant in the posterior end of the cell where cytoskeletal assembly takes place. It may be that this allows for a relatively dynamic region of the cytoskeleton where microtubule assembly can occur. Other proteins associated with the cytoskeleton have defined roles. BILBO1 is a component of the flagellar collar, a structure that is associated with the cytoskeleton near the opening of the FP (Bonhivers et al., 2008). In the absence of BILBO1, cells are unable to undergo cytokinesis and a zipper-like mechanism for synthesizing the FAZ in the daughter cell cannot be initiated. This results in a detached flagellum that is connected to the cytokinesis-arrested cell body only at the base of the flagellum.

Taken together, this shows that even with a robust structure, the *T. brucei* cytoskeleton has a high degree of plasticity in both form and function to allow for changes in cellular architecture.

### Cellular division in *T. brucei* procyclic trypomastigotes

**Figure 2:**
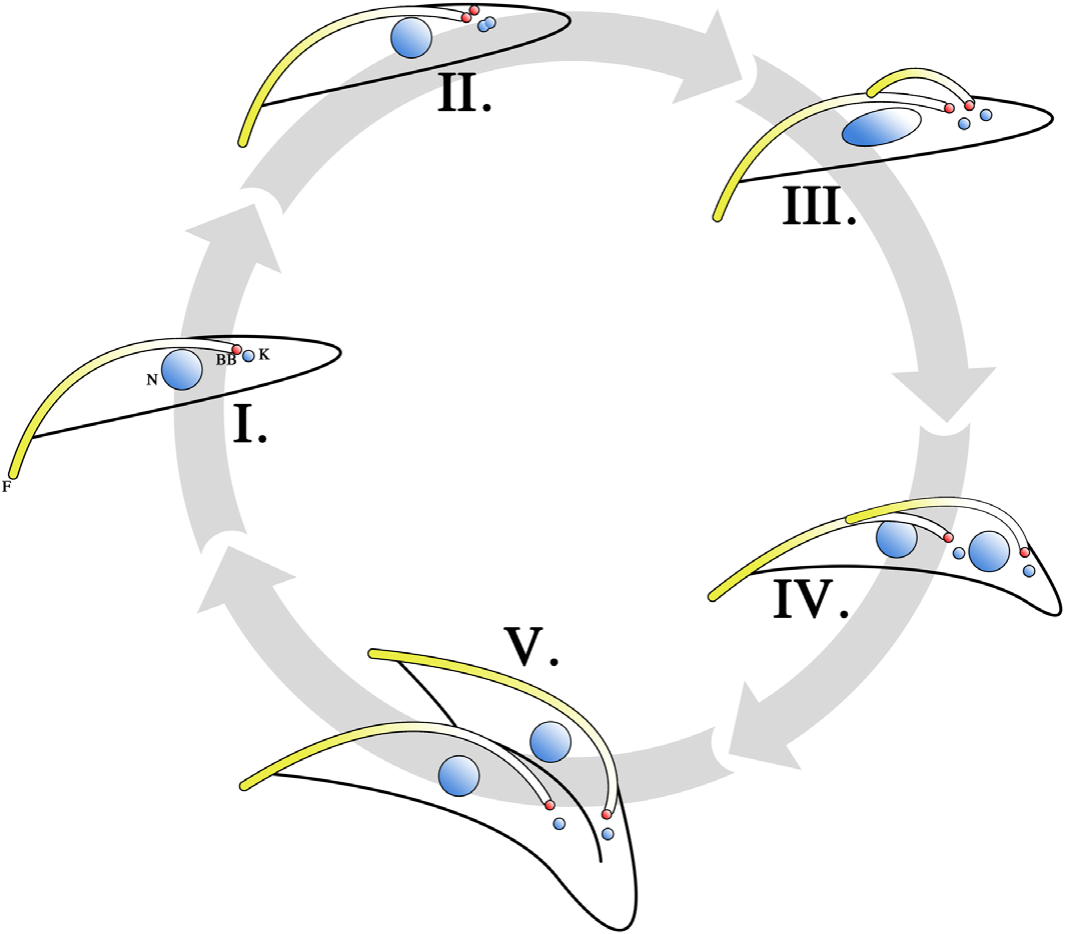
**Cell cycle of *T. brucei* procyclic trypomastigotes.** Cells in the G_1_ phase are in the 1 kinetoplast, 1 flagellum, 1 nucleus (1K1F1N) configuration, with the basal body (BB) in red (I). Upon entering the S phase, nuclear and mitochondrial DNA begin to replicate (II). This can be observed as a change in the size and shape of the kinetoplast. The pro-basal body matures into second basal body and growth of the daughter flagellum is initiated from the new basal body. When growth of the daughter flagellum reaches the point where its tip exits the flagellar pocket, an invagination at the base of the parent and daughter flagellum occurs to form a new flagellar pocket. The daughter flagellum is directed to be in contact with the parent flagellum at its distal tip. The contact point between the distal tip of the daughter flagellum and the parent flagellum is marked by a structure termed the flagellar connector (FC). The growth of the daughter flagellum counteracts the tethering effects of the basal body by the flagellar attachment zone (FAZ) and pushes the daughter basal body (and hence the kinetoplast) away from the parent basal body. Therefore, the cell enters its G_2_ phase in a 2K2F1N configuration (III). Upon entering M phase, the division of the nucleus becomes apparent (IV) and the cell is in the 2K2F2N configuration. The FC is subsequently lost and the daughter flagellum is then allowed to beat freely. The mechanical forces generated by this beating is thought to facilitate cytokinesis (V). Cell cleavage is initiated at the anterior point of dividing cell and follows the FAZ toward the parent basal body. Completion of cytokinesis gives rise to 2 daughter procyclic trypomastigote cells.

A method allowing for the proliferative growth of procyclic *T. brucei* in culture was first described more than 30 years ago (Brun and Schonenberger, 1979). Since then, extensive investigations into the cell cycle of cultured *T. brucei* procyclic trypomastigotes have been carried out. As in other eukaryotic cells, G_1_-, S-, M- and G_2_-phases are present where chromosome replication, kinetoplast segregation, nuclear division and cytokinesis occur over defined periods. Typical of eukaryotic organisms, commencement of the stages and the fidelity of the processes involved in the cell cycle are mediated by a series of kinase cascades, though compared to other eukaryotes, these cascades are relatively streamlined in *T. brucei* (Kumar and Wang, 2006; Rothberg et al., 2006; Umeyama and Wang, 2008; Li et al., 2009). The cell division process (Figure 2) begins with a cell in the G_1_ stage (Figure 2I), where a non-dividing parasite has 1 kinetoplast, 1 flagellum and 1 nucleus (1K1F1N). DNA replication is initiated when the parasite enters the S phase of the cell cycle, both in the kinetoplast and in the nucleus (Figure 2II). This is evident from the increase in size and change in shape of the kinetoplast, as well as the change in shape of the nucleus and the formation of cohesin complexes that hold replicated sister chromatids together (Sharma et al., 2008). The pro-basal body, which lies beside the parent BB, matures and forms a daughter BB. Both BBs acquire a pro-basal body before the daughter flagellum proceeds to elongate from the daughter BB (Gluenz et al., 2011). A new FP is formed as the daughter flagellum breaches the opening of the FP (Lacomble et al., 2010). The extending daughter flagellum is directed toward the parent flagellum and a flagellar connector (FC) is formed between the distal tip of the daughter flagellum and the parent flagellum (Moreira-Leite et al., 2001; Briggs et al., 2004). The FAZ for the daughter flagellum forms between the FC and the base of the flagellum. This serves to tether the daughter BB in position. This tethering is counteracted by both the growth and beating of the daughter flagellum and the daughter BB is pushed toward the posterior of the cell (Absalon et al., 2007). It is through this mechanism that the daughter kinetoplast, which is connected to the daughter BB (Ogbadoyi et al., 2003), is seen to segregate from the parent kinetoplast. This is a phenomenon that is not observed in cell lines that do not construct a FAZ, such as in the case of BILBO^RNAi^ cell lines (Bonhivers et al., 2008), as the absence of FAZ filaments eliminates the anchoring of the BB and hence would not necessitate the elongation of the daughter flagellum to negate this tethering effect. The process of pro-basal body maturation and segregation has been shown to be under the mediation of a polo-like kinase (Hammarton et al., 2007).

The dividing procyclic trypomastigote enters the G_2_ phase with a 2K2F1N configuration (Figure 2III). The chromosomes continue to segregate and cohesin proteins connecting sister chromatids are lost as the cell enters the M phase of its cell cycle (Sharma et al., 2008). At this stage the cell is in the 2K2F2N configuration (Figure 2IV). The FC is subsequently lost (Briggs et al., 2004) and the fully formed daughter flagellum is allowed to beat unhindered. Cytokinesis (Figure 2V) is initiated at this stage and cell cleavage begins at the anterior end of the daughter cell following the FAZ (Robinson et al., 1995; Kohl et al., 2003). It is thought that the mechanical forces generated by the beating parent and daughter flagella aid cell division. This is supported by observations that both flagellar-beat deficient mutants (Branche et al., 2006; Ralston et al., 2006) and cells lacking a flagellum (Kohl et al., 2003; Absalon et al., 2008) have a longer doubling time compared to cell populations that do not have a flagellar defect. Cell growth approximating that of non-mutant trypanosomes can be restored when these cultures are grown under conditions where the culture flask is subjected to continuous shaking (Branche et al., 2006; Ralston et al., 2006). This suggests that shear forces generated by beating flagella helps cytokinesis to proceed.

During the cell cycle, there is incorporation of tubulin subunits into the cytoskeleton at the posterior end of the dividing cell, as well as at the distal tip of the daughter flagellum as it grows. Experimental evidence has shown that the process of cellular elongation at the posterior end may be regulated at the RNA level, as RNA-binding proteins have been associated with the formation of a “nozzle” phenotype where abnormal cellular elongation at the posterior end of the cell occurs. *T. brucei* zinc-finger (TbZF) proteins, with RNA binding motifs, cause the “nozzle” phenotype when they are ectopically overexpressed (Hendriks et al., 2001), while ALBA proteins do the same when they are suppressed by RNA inhibition (Subota et al., 2011). This suggests that TbZF and ALBA proteins represent 2 aspects of a regulation machinery at the RNA level with which microtubule elongation at the posterior of the cytoskeleton is mediated. *T. brucei* kinesins, particularly TbKINC and TbKIND are also thought to work in tandem to mediate the elongation of the posterior end of the cell (Hu et al., 2012a,b). It has been proposed that one possible way through which they could achieve this is by helping to position nucleation centers for tubulin incorporation into the cytoskeleton.

The daughter flagellum is constructed by IFT, a series of molecular complexes that travel to and from the tip of the flagellum driven by kinesin and dynein motors. Shown to carry components of the flagellar axoneme to the distal tip of *Chlamydomonas* flagella (Qin et al., 2004), the silencing of these IFT trains in *T. brucei* results in the parasite failing to construct the daughter flagellum (Kohl et al., 2003; Davidge et al., 2006; Absalon et al., 2008). It therefore stands to reason that IFT plays a similar role in axonemal subunit transport in *T. brucei* flagellar construction. Curiously, although IFT in dividing *T. brucei* procyclic trypomastigotes is constitutive in the old and new flagellum (Absalon et al., 2008), only the flagellum of the daughter cell elongates during cell division. This suggests that the cell can modulate IFT in a stage specific manner, transferring material to and from the flagellum according to cell cycle requirements. This notion is further supported by the observation that cell lines unable to properly construct the PFR show a stage specific accumulation of PFR material only at the distal end of the new flagellum (Bastin et al., 1999), a phenomenon which is thought to be IFT mediated. The growth of the flagellum contributes significantly to the length and shape of the cell, and this has been observed to be true either *in vitro* in cultured cells (Kohl et al., 2003), or *in vivo* in the tsetse (Sharma et al., 2008; Rotureau et al., 2012). This is because the length of the flagellum influences the elongation of the FAZ (Kohl et al., 2003), which has been shown to be the determining factor on daughter cell length as RNAi cell lines able to construct a full length flagellum but not a FAZ results in daughter cells of significantly reduced length (Zhou et al., 2011).

**Figure 3:**
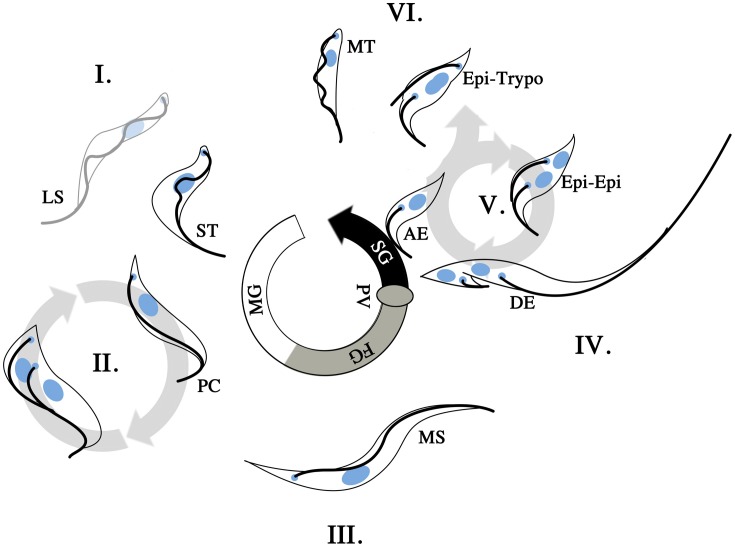
***T. brucei* life cycle in the tsetse.** *T. brucei* undergoes morphological changes as the infection of the tsetse progresses. Upon uptake of an infected bloodmeal, long slender (LS) and short stumpy (ST) bloodstream form trypomastigotes are present in the midgut (I). The ST trypomastigotes will differentiate into procyclic trypomatigotes (PC) to continue the life cycle while the LS trypomastigotes will not. The PC forms are proliferative (II) and will proceed to establish the infection in the midgut of the tsetse. This generates a reservoir of infective parasites in the midgut of the fly. At some point in the infective process (>3 days post infection), the parasites cross into the ectoperitrophic space and proceed to migrate anteriorly along the tsetse alimentary canal. As the infection progresses longer mesocyclic trypamastigotes (MS) are found in the vicinity of the foregut (FG; III). This change in parasite length is presumably an adaptation to a more migratory stage in the life cycle. The MS parasites reenter the lumen of the gut at the proventriculus (PV), where epimastigote and subsequently asymmetrically dividing epimastigote (DE) forms are found. The DE forms give rise to a short epimastigote and a long epimastigote. Should the parasite population successfully establish itself in the salivary glands (SG), epimastigotes are found to be attached (AE, attached epimastigotes) to the SG epithelium and proceed to proliferate via the Epi-Epi cycle (V). The Epi-Epi cycle allows the AE forms to establish an infective population within the SG. Later on in the infection of the SG, Epi-Trypo cell division occurs, which gives rise to trypomastigote form parasites. These trypomastigotes are thought to subsequently form the infective metacyclic trypomastigote (MT) population in the SG. When a SG infected tsetse feeds on its subsequent vertebrate host, MT cells are then injected at the site of feeding where they are allowed to attempt the vertebrate infective process.

Procyclic trypomastigote cell division occurs in the midgut of the tsetse (Figure 3II) to generate a pool of parasites that can then attempt the task of infecting the tsetse salivary glands (SG). The major cell cycle events leading up to cytokinesis in midgut procyclic trypomatigotes appear to be very similar to those observed *in vitro* (Sharma et al., 2008). The other known example of proliferative division is in the attached epimastigotes inhabiting the tsetse SG as a means to colonize the SG of the tsetse (Figure 3V). Though the relative positions of the nucleus and kinetoplast are different in attached epimastigotes, images taken during detailed studies suggest that symmetric cell division in these cells follow a very similar sequence of events (Rotureau et al., 2012).

The passage of *T. brucei* within the tsetse is not restricted to proliferative cell division events. Asymmetric division to generate long and short epimastigotes in the proventriculus (PV) and the epimastigote to trypomastigote stage in the SG are part of the series of complex morphological changes *T. brucei* goes through to complete its life cycle. Certain aspects, such as the relative positions of the kinetoplast and nucleus, are different in these asymmetric division steps. However, the sequence of key events such as the growth of the daughter flagellum, the segregation of the daughter kinetoplasts, nuclear division, and cytokinesis resembles that observed in procyclic trypomastigotes. Hence the cell cycle of cultured procyclic trypomastigotes is a convenient base with which to better understand the more complex events involved in the life cycle of *T. brucei* within the tsetse.

### The trypanosome in the tsetse

As life threatening and dilapidating as AAT and HAT are, *T. brucei* is unable to infect a new vertebrate host without transmission via the bite of an infective tsetse. Beyond basic interests in biology, much attention has been devoted to the life cycle of *T. brucei* within the tsetse in a bid to discover novel methods for containing AAT and HAT. *T. brucei* has the most complex life cycle in the tsetse amongst all the African trypanosomes and traverse the most biological barriers to finally achieve vertebrate infectivity in the SG of the fly (Figure 3). *T. brucei* undergoes changes in cell length and width, flagellar length, and the relative positions of the parasite nucleus and kinetoplast as it traverses the tsetse (Hoare and Wallace, 1966; Sharma et al., 2008). The kinetoplast and nucleus switch positions relative to one another as a *T. brucei* infection progresses in the tsetse.

*T. brucei* infection of the tsetse starts out when BSF trypomastigotes are ingested into the midgut of the tsetse (Figure 3I), with the short stumpy (ST) BSF trypomastigote subpopulation induced to transform into procyclic trypomastigotes that are proliferative in the tsetse midgut (Gibson and Bailey, 2003). The long slender (LS) BSF trypomastigotes are unable to undergo transformation to tsetse-infective forms, and presumably die out in the tsetse midgut. The procyclic trypomastigotes proceed to replicate (Figure 3II) and infect the midgut of the tsetse. This proliferative process is subject to a series of bottlenecks where a multitude of factors (tsetse-immunity related or otherwise) can result in the failure of infection (Gibson and Bailey, 2003; Oberle et al., 2010). Should the infection of the midgut be successful, procyclic trypomastigotes will be found in the ectoperitrophic space from day 3 of infection. The infection then proceeds toward the PV at the anterior end of the tsetse alimentary canal. This forward migration is accompanied by the appearance of long mesocyclic trypomastigotes (Figure 3III) that are thought to facilitate migration with their elongated flagellum. At the PV, the parasites proceed to reinvade the lumen of the alimentary canal and it is in this organ that long epimastigotes can first be observed. The long epimastigotes then proceed to asymmetrically divide into long and short epimastigotes (Figure 3IV), and this is hypothesized to facilitate the delivery of the short (and thought to be poorly motile) epimastigote to the SG by the long (and thought to be more highly motile) epimastigote (Van den Abbeele et al., 1999). This appears to be a second infective bottleneck for *T. brucei* in tsetse, with only a fraction of tsetse with infected midguts harboring parasites in their SG (Peacock et al., 2012). The attachment of epimastigotes upon the epithelium of the tsetse SG is an indication that the second bottleneck has been circumvented. These SG attached epimastigotes are proliferative and proceed to colonize the SG via cell division (Figure 3V). Later in the infection of the SG, another example of asymmetric division is initiated in the SG epimastigote population, where the epimastigotes undergo epi-trypo cell division (Figure 3VI) to give rise to trypomastigotes that are thought to be the progenitors for the metacyclic form in the SG (Rotureau et al., 2012). Once a population of metacyclic parasites is established, the cycle can then begin anew when the infected tsetse feeds on a vertebrate host.

**Figure 4:**
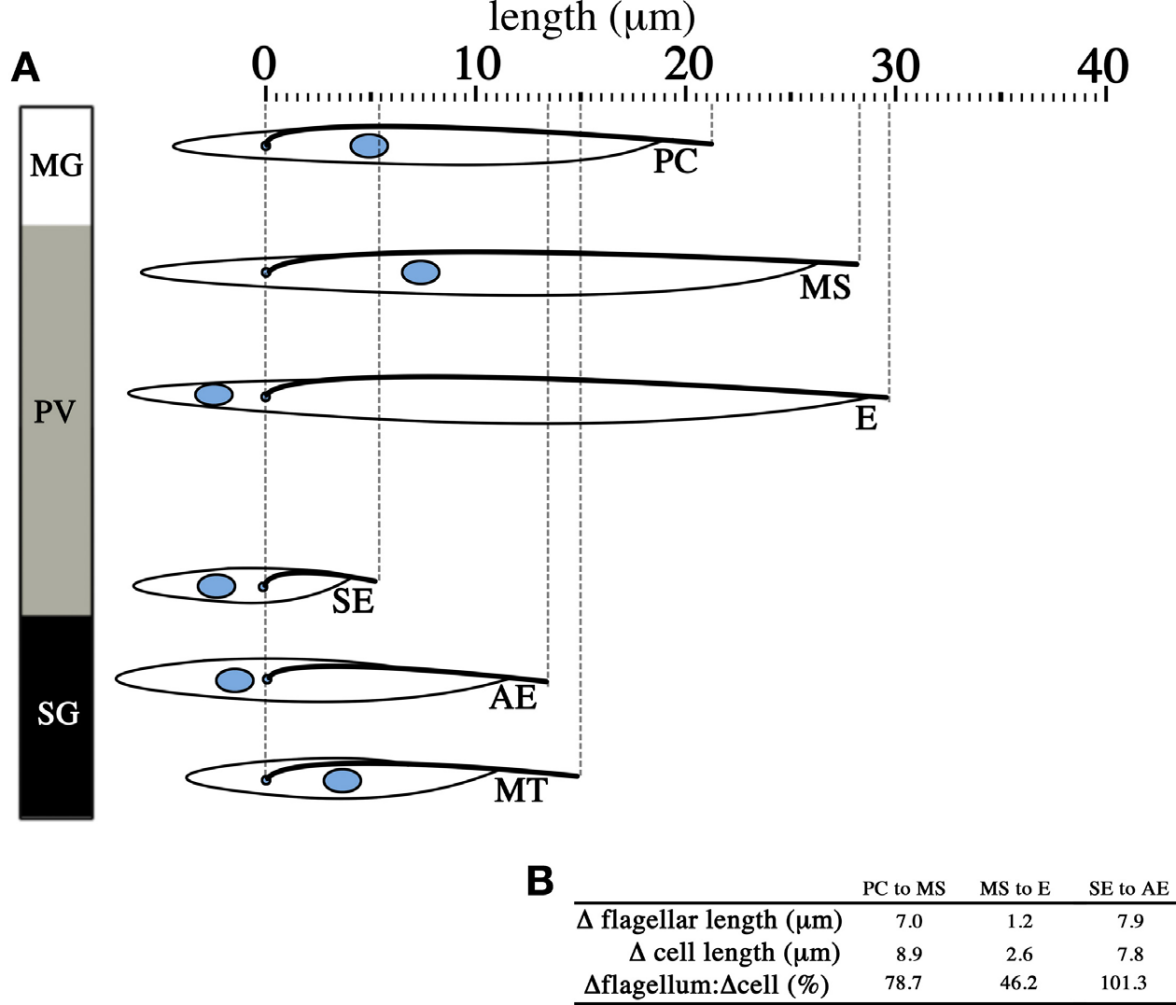
**Change in flagellar length contributes significantly to changes in cell length.** The diagram lines up parasites from different infective stages in the tsetse with the cell posterior to the left and the cell anterior to the right. *T. brucei* undergoes significant changes in cell length **(A)** across the life cycle stages found in the midgut (MG), the proventriculus (PV) and the salivary glands (SG). When life cycle stages that arise independent of asymmetric division are considered **(B)**, the increase in length of the flagellum (Δflagellar length) from one life cycle stage to the subsequent one is significant and in most cases make up the majority of the change in total cell length (Δcell length). Only the measurements for the distance of the nucleus to the cell posterior, distance between the nucleus and the kinetoplast, flagellar length and total cell length are reproduced from published data (Rotureau et al., 2011). PC = procyclic trypomastigote, MS = mesocyclic trypomastigote, E = epimastigote (proventricular), SE = short epimastigote, AE = attached epimastigote, MT = metacyclic trypomastigote.

These easily observable morphological changes are accompanied by spectacular physiological and biochemical changes. These include the switching of the surface coat consisting of variant surface glycoproteins (VSGs) to procyclins and eventually to BARPs, which were historically named bloodstream alanine-rich proteins prior to the discovery of their association with SG epimastigotes (Roditi et al., 1989; Urwyler et al., 2007), as well as the switch from glucose-dependence in BSFs to proline-dependence in tsetse forms for cellular metabolism. These changes are extensively reviewed elsewhere (Matthews, 1999; Walshe et al., 2009). Interestingly, the parasite flagellum appears to contribute significantly to the elongation of the cell during the life cycle in the tsetse (Figure 4), and in the instance of the transition from short epimastigotes in the PV to the attached epimastigotes in SG, the increase in flagellar length compared to the preceding short epimastigote form is actually greater than the total increase in cell length (Figure 4B). This is perhaps reflective of the multiple important functions of the parasite flagellum during the tsetse infection cycle, such as motility for migration, adhesion to the SG epithelium (Vickerman, 1985), and as potential environmental sensors (Rotureau et al., 2009; Oberholzer et al., 2010).

*T. brucei* has also been documented to undergo meiosis and genetic exchange at the SG stage of tsetse infection. This was first demonstrated indirectly in tsetse infected with mixed strains of trypanosomes where enzymatic and genetic differences in parasites that were subsequently harvested from the tsetse suggested a mixing of traits from parental strains (Jenni et al., 1986). Two decades later, transgenic trypanosome cell lines expressing different fluorescent proteins in the cytosol resulted in cells expressing a mixed repertoire of fluorescent proteins being found in the SG in tsetse where infections were allowed to mature, directly demonstrating genetic exchange in the parasites (Peacock et al., 2008). With the use of fluorophore-fused proteins, meiosis-associated proteins were subsequently shown to be expressed in SG epimastigotes, thus confirming the existence of a meiotic stage in tsetse (Peacock et al., 2011).

This diversity of forms involved in the *T. brucei* life cycle highlights the demands of plasticity on a cell structure that has evolved to be highly robust. In the tsetse, *T. brucei* faces a series of migrational challenges during the infection process and have to overcome a multitude of biological barriers, such as traversing the peritrophic matrix, to finally achieve infectivity in the tsetse SG (Peacock et al., 2007). Proteases are present in the posterior midgut of the tsetse, against which the procyclin coat is thought to provide a degree of protection (Liniger et al., 2003; Vassella et al., 2009). Furthermore, variations across the biological compartments, such as osmolarity or pH (Liniger et al., 2003) likewise necessitates a cellular structure that is resistant to damage when the parasite is in the tsetse.

## Molecular mechanisms for cytoskeletal pasticity

Due in part to its role as a model organism, our understanding of *T. brucei* cell biology is backed by a respectable body of published data. With regards to *T. brucei* in the tsetse, this understanding has largely been influenced by what has been experimentally feasible on trypanosomes, with some of it having to be inferred from work done on other organisms. This is by no means an attempt at downplaying the considerable ingenuity and effort that has gone into investigating a biological relationship that is extremely complex and at times elusive, but rather a statement of caution that our understanding of the molecular mechanisms mediating trypanosome morphology in the tsetse is far from complete.

### Organelle translocation by microtubule-mediated sliding?

The translocation of cellular components is an integral part of the *T. brucei* life cycle. The more easily observable of these events include the changing of the relative positions of the kinetoplast and nucleus between epimastigotes and trypomastigote forms in the tsetse. Based on the observations and measurements taken of parasites in the tsetse (Sharma et al., 2008; Rotureau et al., 2011), it appears that neither the kinetoplast nor the nucleus is static when this translocation process occurs. The anterior movement of the kinetoplast (away from the posterior end of the cell) has been shown to be a cumulative result of the posterior end elongating and the migration of the kinetoplast toward the anterior of the cell (Matthews et al., 1995), at least in the initial stages of infection as ST BSF trypomastigotes transform into procyclic trypomastigotes. However, it is apparent that the nucleus likewise translocates toward the posterior end of the cell during the formation of long epimastigotes in the PV as the nucleus is not only in a more posterior position compared to the kinetoplast, but also closer to the posterior of the cell (Figure 4A). This decrease in distance of the parasite nucleus to the posterior end of the cell may be the result of cytoskeleton destabilization and shortening at the posterior end, or the movement of the nucleus to a more posterior position in the cell.

Microtubule-depolarizing kinesins (of the kinesin-13 family) have been implicated in the shortening of flagellar and mitotic microtubules in other flagellated protozoans such as *Giardia, Chlamydomonas* and *Leishmania* (Blaineau et al., 2007; Dawson et al., 2007; Piao et al., 2009). Although *T. brucei* kinesin-13 family proteins retain the microtubule depolymerization motif and have been suggested to have multiple roles in the cell (Wickstead et al., 2010), the only knockdown study involving a member of the kinesin-13 family in *T. brucei* was shown to only impair mitosis by affecting chromosomal segregation without having an effect on the flagellum (Chan et al., 2010). It is of course premature to discount a role for *in situ* microtubule disassembly in either the flagellum or the cytoskeleton with the other *T. brucei* kinesin-13 family of proteins, but based on the current understanding of how the parasite shortens its cellular and flagellar length in the tsetse (via asymmetric division), it is unlikely that the translocation of the parasite nucleus close to the posterior end of the cell is the result of cytoskeleton disassembly.

The translocation of the nucleus has been demonstrated to be under the regulation of ALBA RNA binding proteins. Simultaneous RNA inhibition of ALBA3 and 4 in cultured *T. brucei* procyclic trypomastigote results in cells with a nucleus posterior to the kinetoplast, while ALBA 3 overexpressing cells are able to undergo all the morphological changes leading up to the long epimastigote stage in the PV but are unable to translocate their nucleus toward the posterior of the cell (Subota et al., 2011). Taken together, these observations suggest that the binding of ALBA proteins to RNA inhibits the translocation of the nucleus and kinetoplast, a state that can only be reversed when ALBA binding no longer occurs.

This relative positioning of the kinetoplast and nucleus is again reversed in the metacyclic trypomastigote stages in the tsetse SG, and the question is what subcellular mechanisms allow for this dynamic repositioning of organelles in *T. brucei*? In other biological systems, it has been determined that organelle translocation in the cell is mediated by kinesin and dynein motors that drive the migration in a way that is dependent on the polarity of the microtubule scaffold (Rogers et al., 1997; Tuma et al., 1998; Goldstein and Yang, 2000; Lee et al., 2011; Baumann et al., 2012). Kinesin motors mainly drive movement from the “−”to “+” ends, and dynein motors drive movement in the opposite direction. The microtubules in the *T. brucei* cytoskeleton are arranged in such a manner that they have a polarity with the “+” end at the posterior end and the “−” polarity at the anterior end (Robinson et al., 1995), though this polarity is reversed in the flagellum and the microtubule quartet of the FAZ (the “−” end at the proximal base and the “+” end at the distal tip). Therefore, the microtubule scaffold is compatible with motor protein-mediated organelle sliding, the next logical question is whether there is evidence of these motor proteins present in *T. brucei*?

Relatively little is known regarding how microtubule-directed motors would mediate organelle translocation in the cell body of the parasite. Recently 2 *T. brucei* kinesins, TbKinC and TbKinD, were shown to work in tandem to maintain the stability of the cytoskeleton (Hu et al., 2012a,b). Although RNAi experiments on TbKinC and TbKinD led to a cessation of cell division and aberrant microtubule corset arrangement in affected cells, the nucleus and kinetoplasts still appeared to divide and segregate. This does not completely eliminate kinesin-mediated organelle transport as being responsible for kinetoplast-nucleus translocation of the type observed in trypomastigote to epimastigote (Epi-Trypo) transformations, since as many as 46 kinesins are present in the *T. brucei* genome (Berriman et al., 2005).

In other eukaryotic cellular systems, nucleus migration is associated with either actin or tubulin scaffolds. The nucleus is connected to these scaffolds by Sad1p/UNC-84 (SUN) and klarsicht/ANC-1/Syne homology (KASH) proteins that form the LINC (linker of the nucleoskeleton to the cytoskeleton) complex, a structure whose characteristics are extensively reviewed elsewhere (Tapley and Starr, 2013). The LINC complex has been documented to allow the nucleus to harness the movement of retrograde actin cables for translocation in fibroblast cells (Luxton et al., 2010; Folker et al., 2011; Borrego-Pinto et al., 2012), as well as undergo bidirectional movement in the hypodermal cells of *Caenorhabditis elegans* embryos (Fridolfsson and Starr, 2010). A rudimentary BLASTp search of TriTrypDB (Aslett et al., 2010) for LINC complex proteins, thought to be conserved across eukaryotes, yielded proteins of varying sequence identity (25–44%) across several trypanosome species. Whether LINC complexes are involved in the bidirectional translocation of the nucleus in *T. brucei* is unknown at this point. Future investigations may shed more light on this unresolved issue.

Daughter kinetoplast segregation in procyclics has been shown to be a mechanical process of push and pull between the extending daughter flagellum and the FAZ filaments (Absalon et al., 2007) and may be independent of such a molecular motor-driven system. Perhaps a more appropriate experimental design to assay the mystery of kinetoplast-nucleus translocation in Epi-Trypo transformations may be to carry out knockdowns of molecular motors on transforming cells, but this has to date been proven technically challenging with regards to obtaining sufficient biological material for such an experiment. Kinetoplast-nucleus migration is a stage specific process for *T. brucei* in the tsetse. While kinesin-dynein interactive arrangements have been shown to be present in other cellular systems where temporally regulated translocation of organelles is required (Wubbolts et al., 1999), it remains unclear if such an arrangement occurs in *T. brucei*.

### Morphological variation through asymmetric regulation of flagellar length

*T. brucei* has a complex life cycle in the tsetse, encompassing many stages with very different cellular morphologies. The reasons for having these many life stages are not completely clear, but as the various forms usually coincide with a specific stage of a gradually advancing infection process, variation in form is thought to coincide with requirements in function. The mesocyclic trypomastigotes and long epimastigotes are thought to allow for migrating relatively long distances across multiple biological compartments in the tsetse, while shorter forms usually coincide with a stage in the life cycle which is either proliferative or where motility across relatively long distances is not required (Van den Abbeele et al., 1999; Sharma et al., 2008). Proliferative cell division occurs at the procyclic trypomastigote stage in the midgut (Figure 3II) and at the attached epimastigote stage in the SG (Figure 3V) where daughter cells similar in form to the parent cells are produced. All other canonical life cycle stages are therefore part of a variation in the cell cycle that leads to asymmetric division into 2 unequal daughter cells.

Probing of *T. brucei* parasites collected from the tsetse with antibodies specific for the sister chromatid cohesion component (SCC1) allows for the identification of cells in the S- or G2-phase of the cell cycle (Sharma et al., 2008). SCC1 holds sister chromatids together after chromosome replication in the S-phase of the cell cycle and is maintained until the M phase where the sister chromatids separate. When a heterogeneous population of trypanosome cells is probed for SCC1, it is therefore, possible to elucidate if cells of distinct morphology are independently dividing stages or if they are part of a single cell cycle. SCC1 is present in the nucleus of mesocyclic trypomastigotes in the foregut, the nucleus is elongated but none of these cells are undergoing cell division as evident from the lack of kinetoplast segregation or nuclear division. Loss of SCC1 and kinetoplast segregation only occurs in asymmetrically dividing epimastigotes. This indicates that the mesocyclic trypomastigotes, long epimastigotes, and asymmetrically divided long and short epimastigotes are all part of a single cell cycle, with cellular elongation occurring prior to assymetric division.

**Figure 5:**
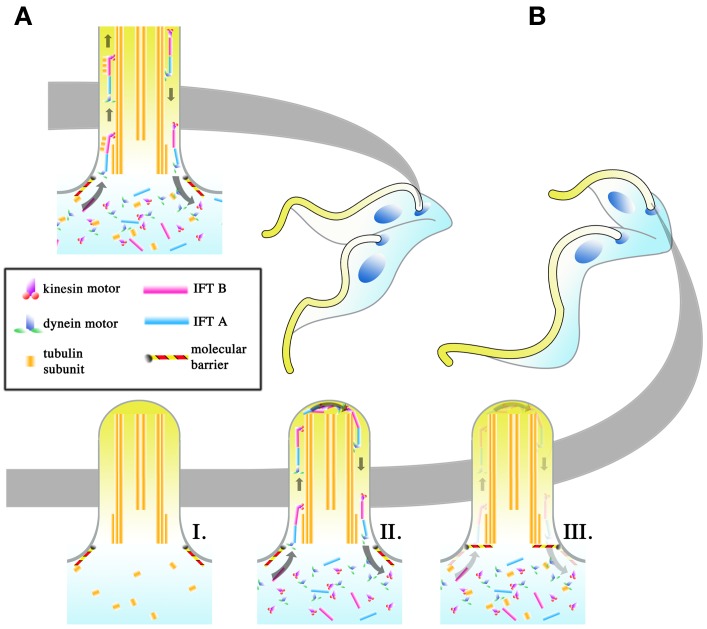
**Regulation of intraflagellar transport (IFT) as a means to change flagellar length.** The regulation of the flagellar length of the daughter cell may be an important means with which *T. brucei* achieves its different infective morphologies in the tsetse. In symmetrically dividing cells **(A)**, IFT is allowed to proceed as normal in the daughter cell and IFT “trains,” made up of IFT A and B complexes and driven by kinesin (base to tip) and dynein (tip to base) motors, are thought to carry flagellar components to the tip of the flagellum where they are used in flagellar construction. In asymmetrically dividing cells **(B)**, 3 potential strategies may be used by the dividing cell to shorten the total length of the flagellum in the daughter cell. The depletion of IFT components (I), regardless if the depletion includes all the IFT components, will halt flagellar construction. Even if the pool of flagellar components is retained, the lack of IFT would make the incorporation of those subunits into the flagellum impossible. Regulation might likewise occur at the level of the flagellar components (II), such as depleting the pool of available tubulin for incorporation into the flagellum. IFT would still occur in the daughter flagellum, though the length of the daughter flagellum cannot be extended without the components to build it with. The placement of molecular barriers (III) would stop the entrance of IFT components into the flagellum. This does not exclude the possibility that the barriers are selective, with IFT trains being allowed to enter the flagellum while structural subunits for construction are retained.

Interestingly, the dimensions of the elongating trypomastigotes (PC to MS) and the long and short daughter epimastigotes (E and SE, Figure 4A) differ most significantly in the length of their flagellum (Figure 4B, for full morphometric measurements, please refer to Table S1 of Rotureau et al., 2011). This observation is in agreement with experiments carried out *in vitro*, where procyclic cells have been shown to undergo flagellar elongation independent of cell division (Farr and Gull, 2009). More strikingly, the asymmetric division that produces the long and short epimastigotes is reminiscent of cell division in RNAi experiments where genes involved in anterograde IFT, which are involved in flagellar base to distal tip transport, are disrupted. As RNAi induction progresses in these experiments, daughter cells with progressively shorter flagellum are produced during cell division. This occurs regardless of the IFT component targeted and always results in the gradual failure to construct a full-length flagellum in daughter cells (Kohl et al., 2003; Davidge et al., 2006; Absalon et al., 2007, 2008). Thus, it is plausible that asymmetrically dividing epimastigotes regulate IFT as a means to regulate the cell length of their short daughter cell (Figure 5). However, although *in vitro* experiments have typically involved the targeting of IFT complex components (a situation similar to Figure 5BI), 2 other models are likewise possible when it comes to restricting flagellar construction in a daughter cell. Depletion of flagellar structural components available to the daughter flagellum may be another way in which construction of the daughter flagellum can be stunted (Figure 5BII). It would be interesting to determine if the depletion of flagellar building material is a convenient consequence of the degree of elongation the parent flagellum undergoes during the mesocyclic trypomastigote stage, where a finite pool of tubulin subunits that is available for both parental flagellum elongation and daughter flagellum construction is depleted when it is time to construct a daughter flagellum. This assumption is yet to be supported by any experimental evidence. Molecular barriers could also be put in place to block entry of IFT components into the flagellum (Figure 5BIII). These barriers could be selective, barring the entrance of structural components of the flagellum while allowing IFT trains to be assembled and carry out their assigned circuits.

Of the 3 proposed models, the depletion of IFT components would result in conditions where IFT trains no longer traverse the flagellum. Given the other proposed roles of IFT beyond flagellar construction (Rotureau et al., 2009; Buisson et al., 2013), it is implausible that IFT would be completely depleted in *T. brucei* within the tsetse, with the barrier model or the structural component depletion model (or both) being the more probable way with which to cut short the construction of the daughter flagellum.

### Molecular triggers for cytoskeletal plasticity

The emergence of specific stages of *T. brucei* usually coincides with fixed biological compartments within the tsetse (Figure 3). For example procyclic trypomastigotes are usually confined to the posterior midgut, mesocyclics are located along the posterior midgut toward the anterior midgut and foregut, asymmetrically dividing epimastigotes are found in the PV while epimastigotes and metacyclic trypomastigotes are found in the SG. This correlation of life cycle stages to tsetse biological compartments may be entirely a parasite-regulated event, or may just as likely involve tsetse-specific signals. Likewise, the cues involved in other phenomenon, such as attached epimastigotes being induced to undergo the Epi-Trypo asymmetric division remain poorly understood and begs the question on whether tsetse-specific external cues or a form of quorum sensing inherent to trypanosomes are responsible for these phenomena.

To exploit external signals to its advantage, the parasite needs to have sensing mechanisms in place. Several examples in literature indicate that *T. brucei* is capable of perceiving signals from its environment. It has been proposed that density-dependent quorum sensing exists in BSF trypanosomes to signal for the parasite population to transform from the proliferative LS BSFs to non-proliferative ST BSFs in the vertebrate host. To date, the use of conditioned media and computational modeling indicate that this relies on a dose dependent exposure to an extracellular stumpy induction factor (Vassella et al., 1997; MacGregor et al., 2011), however the identification of this molecule remains elusive. More direct experimental evidence has shown that ST BSF trypanosomes perceive TCA cycle metabolites once they are in the tsetse midgut via a PAD (Proteins Associated with Differentiation) receptor to begin transforming into procyclic trypomastigotes (Dean et al., 2009). This perception is dependent upon ambient temperature, and demonstrates that *T. brucei* can be sensitive to both chemical and physical changes in its surroundings. Different sections of the tsetse alimentary canal have different chemical properties, for example the presence of proteases in the posterior midgut and not the anterior hindgut, a slight pH gradient in the direction of the foregut (Liniger et al., 2003), and symbiotic bacteria (and their associated metabolic products) in the specialized organ (termed the bacteriome) at the anterior midgut (Weiss et al., 2013). All are plausible candidates as sources for signals that trigger transformation in parasites in the tsetse.

How would these external signals be perceived by the parasite? The FP is a possible candidate as the site where external signals can be introduced into the cell body. It has been shown that PAD receptors, which are distributed across the surface of the cell, accumulate at the FP to be endocytosed during ST BSF to procyclic trypomastigote transformation in the tsetse midgut (Dean et al., 2009). Although proven to be of major importance for parasite survival in BSF parasites, FP mediated endocytosis (and hence the uptake of external molecular signals) has been shown to be of lesser importance for procyclic trypomastigotes grown in culture (Garcia-Salcedo et al., 2004). However, receptor mediated endocytosis nonetheless still occurs in procyclic trypomastigotes in culture, only that its ablation does not lead to cell death *in vitro*. It is unclear whether this lack of dependence on FP mediated endocytosis in procyclic trypomastigotes is an artifact of culturing. In culture, parasites are presented with an environment where nutrients are abundant and transformation beyond the procyclic trypomastigote form is not required for survival. Therefore, this independence of molecular uptake may not hold true for the survival of *T. brucei* fly-infective forms during infection of the tsetse.

An alternative mechanism for extracellular signal perception is via the use of the parasite flagellum as a sensing antenna. This has been extensively reviewed for other biological systems, both unicellular and multicellular (Singla and Reiter, 2006). A mitogen-activated kinase (MAPK) in *Chlamydomonas* has been implicated in helping the protist sense its size prior to initiating mitosis (Bradley and Quarmby, 2005). A series of phosphodiesterases and adenylate kinases (enzymes implicated in signaling pathways) have likewise been identified in the trypanosome flagellum (Broadhead et al., 2006). It has been proposed in a previous review (Rotureau et al., 2009) that these kinases may work in tandem with IFT, where IFT serves to concentrate and dissipate them as needed for signal transduction from the flagellum to the rest of the cell body. Evidence for these hypotheses remains scarce and future experimentation may shed more light on this issue.

## Conclusion: a promising time?

A full larder of molecular tools is available to the investigator for elucidating molecular events within cultured trypanosomes. However, this ample repertoire of molecular tools falls short when the primary problem is rarity of the cell types with which to experiment with. The infection of tsetse by *T. brucei* is notoriously inefficient. Under laboratory conditions, only a proportion of tsetse are susceptible to initial infection in the midgut, and only a small percentage of those infections can result in infective SG (Peacock et al., 2012). This makes studies on rare tsetse-specific stages of *T. brucei*, such as those that only exist in the SG, extremely difficult. This is usually circumvented at a great expenditure of time and materials (both insect and parasites), a challenge that not many laboratories are able to meet.

The discovery that the upregulation of a single RNA binding protein is able to induce the transformation of procyclic trypomastigotes in culture may solve this dilemma. Ectopic overexpression of RNA binding protein 6 (RBP6) resulted in the transformation of cultured WT 427 procyclic trypomastigotes into tsetse-infective forms up to metacyclic trypomastigotes with VSG coats (Kolev et al., 2012). The resulting metacyclic trypomastigotes were able to infect laboratory mice. This discovery is made even more extraordinary considering that the 427 *T. brucei* strain is degenerate and typically unable to infect tsetse beyond the midgut stage. There are of course documented exceptions to this rule of degeneracy (Peacock et al., 2008), but this generalization holds true for most 427 strains utilized for *in vitro* investigations.

Now that reasonable amounts of parasite material can be obtained in culture, the molecular tools developed for *in vitro* assays with *T. brucei* procyclic form parasites can finally be applied to rare forms previously only found in the tsetse. RNAi studies, such as those targeting molecular motors (dyneins and kinesins) can shed more light on how organelles migrate as trypomastigotes transform to epimastigotes, just as they can be used to determine the molecular players involved in asymmetric division.

However, even with this landmark discovery, many questions remain regarding the morphological changes of African trypanosomes in the tsetse (Box 1). First and foremost, can the extremely tsetse-canonical forms, such as the Epi-Trypo dividing parasite or the forms involved in meiotic division and genetic mating, be found in RBP6 up-regulated cultures? Secondly, how is the RBP6 related molecular cascade mediated under non-experimental conditions? Is it purely a density-related signaling mechanism inherent in the parasite? Or must the transformation process be triggered by signals originating in the tsetse? Finally, a pool of trypomastigote procyclics is still maintained in cultures where RBP6 is overexpressed, and the emergence of other tsetse-canonical forms appear to be transient. Is this indicative that only a proportion of the procyclic trypomastigote population is receptive to the effects of RBP6?

Box 1 **Outstanding questions regarding post-RNA binding protein 6.**

**Questions**:

1. Can rare tsetse-specific forms be found in RBP6 overexpressing cultures?
2. What signals trigger the RBP6 cascade?
3. Are subpopulations of *T. brucei* predestined to transform into tsetse canonical forms?

In conclusion, the morphological changes that *T. brucei* undergoes in the tsetse are fascinating. Recent discoveries have greatly enhanced our ability to delve into the mysteries of how these morphological transformations occur. However, pressing questions such as whether *T. brucei* detects external signals to trigger morphological changes during infection of the tsetse may have to be answered with the use of the insect vector.

### Conflict of interest statement

The authors declare that the research was conducted in the absence of any commercial or financial relationships that could be construed as a potential conflict of interest.
